# Risk Calculator for Retinopathy of Prematurity Requiring Treatment

**DOI:** 10.3389/fped.2020.529639

**Published:** 2020-09-18

**Authors:** Maria J. Chaves-Samaniego, Mar García Castejón, Maria C. Chaves-Samaniego, Ana Solans Perez Larraya, Jose Maria Ortega Molina, Antonio Muñoz Hoyos, Jose L. García-Serrano

**Affiliations:** ^1^Doctoral Program in Clinical Medicine and Public Health, University of Granada, Granada, Spain; ^2^Department of Ophthalmology, San Cecilio University Hospital, Granada, Spain; ^3^Bachelor of Medicine, University of Granada, Granada, Spain; ^4^Department of Paediatrics, University Hospital Virgen de las Nieves, Granada, Spain; ^5^Department of Paediatrics, San Cecilio University Hospital, Granada, Spain

**Keywords:** retinopathy of prematurity, retinal vessel, oxygen-induced retinopathy, risk factors, bronchopulmonary dysplasia

## Abstract

**Importance:** Vascular delay that occurs early in the development of retinopathy of prematurity (ROP) is a risk factor that can be compensated by ensuring a good rate of retinal vascularization to avoid ROP that requires treatment.

**Background:** The objective of the present study was to determine the association between ROP that requires treatment and risk factors such as the extent of the temporal avascular area of the retina and the number of days of mechanical ventilation (MV).

**Design:** Observational retrospective case-control study.

**Participants:** Two hundred and twenty-eight premature newborns included in the screening protocol for retinopathy of prematurity.

**Methods:** Subjects underwent retinal examination in the 4 and 6th postnatal weeks.

**Main Outcome Measures**: The temporal avascular area was measured in disc diameters (DD), while the MV time was measured in days of treatment.

**Results:** Patients with a longer MV time had a higher risk of treatment (*R*^2^: 24.7, *p* < 0.0001; increase in risk of 8.1% for each additional day), as did those who showed greater avascular area (*R*^2^: 24.7, *p* < 0.0001; increase in risk of 111% for each additional DD). An online calculator system and a table are presented for calculating the risk of ROP requiring treatment as a function of these two risk factors.

**Conclusions and Relevance:** The temporal avascular area of the retina and MV time must be taken into account in the first examination of the newborn to predict the need for ROP treatment.

## Introduction

Retinopathy of prematurity (ROP) is currently the world's second leading cause of preventable child blindness (1, 2). For this reason, numerous studies have been carried out to identify the main risk factors for ROP (3) and incorporate them into early ROP detection algorithms (4). Current screening algorithms are mainly based on birth weight and gestational age (3). However, several other related factors are involved, such as the avascular area of the retina, the rate of retinal vascularization, and the time spent by the premature newborn under mechanical ventilation (MV) (3–6).

Various ROP predictive models to identify newborns requiring ophthalmologic screenings are reported in the literature. Majority show high sensitivity and medium to low specificity (7–10). Authors themselves have made clarifications regarding their own models, claiming that no predictive model will work properly at a universal level (11); in many cases newborns of ≥31 weeks or <24 weeks of pregnancy, who quite often show a wide area of non-vascularized retina after birth, are excluded (12). However, there is not a single model of practical applicability and adaptability currently available in the usual clinical practice (13) that helps physicians identify newborns with higher risk of severe ROP requiring treatment and guides the planning of checkup frequency.

The DIGIROP model concluded that one of the best variables for predicting risk of requiring ROP treatment is postnatal chronologicalage (12). Conversely, the Early Treatment for Retinopathy Of Prematurity Study showed that the wider the avascular area (zone I), the higher the risk of requiring treatment (14). The more severe a ROP is, the larger the peripheral area of retinal ischemia is, and this is associated with a greater need for revascularization and recovery time (15). Specifically, the revascularization time required to reverse ROP in zone I is significantly longer than that required in zone II, which in turn is longer than that required in zone III (16, 17). A retinal vascularization rate of <0.5 disc diameters (DD) per week (18) has been identified as an indication for ROP treatment (5). Therefore, why not study the temporal avascular area of the retina as a risk factor?

Both bronchopulmonary dysplasia and the need for MV in the premature newborn (19) are closely related to ROP. Longer MV times are associated with greater risk of ROP requiring treatment (6, 20). Recently, new oxygen therapy control measures have been implemented in neonatal intensive care units (NICUs) (21), as has early treatment by intravitreal bevacizumab or laser diode. Both these techniques have contributed to an improved prognosis for newborns with ROP (22).

The objective of the present study was to determine the risk factors for ROP requiring treatment, and to develop a system for calculating the risk of ROP requiring treatment that is based on the main risk factors and can be applied in clinical practice at the first examination of the newborn.

## Materials and Methods

The present study was approved by the Biomedical Research Ethics Committee of Andalusia and was carried out in accordance with the ethical principles of the Declaration of Helsinki for medical research. All medical data were collected anonymously after informed consent was given and after the parents or legal guardians of the study participants gave express authorization.

### Subjects of the Study

An observational, retrospective case-control study was conducted on 228 premature newborns who were examined between 1999 and 2019 at the NICU of the San Cecilio University Hospital in Granada, Spain. One eye of each patient was included in the study.

Inclusion criteria were: (1) birth weight <1,500 g or GA ≤ 32 weeks, (2) birth weight between 1,501 and 2,000 g and GA ≥ 32 weeks with oxygen supply > 72 h or unstable clinical course as determined by the NICU neonatologist (apneas, neonatal acidosis, twin death, intraventricular hemorrhage, persistent ductus, sepsis, necrotizing enterocolitis, or concurrent surgical interventions), (3) preterm newborns that could be examined between the fourth and sixth postnatal weeks, and (4) preterm newborns with at least three examinations performed. Participants were excluded from the study when (1) they could not be examined between the 4 and 6th weeks after delivery, (2) they underwent fewer than three examinations, (3) they had complete retinal vascularization in the first examination, and (4) they showed medial opacity.

### Eye Examination

All patients were examined by the same pediatric ophthalmologist between the 4 and 6th postnatal weeks. The examinations used indirect ophthalmoscopy with indentation and were carried out under pharmacological mydriasis with topical anesthesia using a 20 diopter lens. This technique provides an approximate field of vision of 8 DD, which is equivalent to 45°. First, estimation of the vascular caliber is carried out and presence or absence of dilation or vascular tortuosity found in the vessels of the central retina is evaluated. Subsequently, the ROP stage and compromised area are identified. Next, eyeball indentation is performed in order to observe the peripheral retina by aligning the binocular ophthalmoscope until the raised gray line corresponding to the indentation is visible. Indentation is moved in parallel until the first temporal vessel is visualized in the periphery.

In each examination, the following data were collected: ROP stage according to the International Classification of Retinopathy of Prematurity (16), affected retinal area (zones I, II, and III), time extension, presence of preplus and plus disease, and extent of retinal avascular area (in DD) from the periphery to the center of the retina. Examinations were performed weekly or every 2 weeks until the retina was completely vascularized, except in patients with preplus or plus disease, in those with limited vascularization in zone I or posterior zone II, or in those with stage 3 ROP in any zone. In such cases, examination was carried out weekly.

Eye selection for the study (right or left) was carried out through random allocation.

The need for ROP treatment was recorded as a dependent variable, while the following parameters were recorded as independent variables: temporal avascular area of the retina in DD, GA (weeks), and MV time (days). We also collected weight gain in the first 4 weeks of postnatal life (g/day), Apgar score (23), degree of bronchopulmonary dysplasia (19), number of blood transfusions received, presence of apnea (24), persistence of arterial ductus, presence of sepsis (25), presence of necrotizing enterocolitis, presence of intraventricular hemorrhage and presence of periventricular leukomalacia.

Disc diameters: The measurement unit used to quantify non-vascularized area of the retina was the disc diameter (DD). The horizontal diameter of the subject's optic nerve can be quickly and easily identified during fundus exploration. Once the optic disc diameter is known, extension of any retinal territory can be quantified in DDs. This measurement unit has been used by several authors to quantify the territory of retinal immaturity (26, 27).

Mechanical ventilation time (MV) was defined as the treatment time using endotracheal intubation. MV time was measured in days. However, between 2009 and 2012, the oxygen supply protocols were modified. Saturation levels were maintained at ~95% during the years 2000–2009; from 2009–2012 they varied between 85 and 93% (6).

Postnatal weight gain was defined as the weight gained from birth up to week 4. It is expressed in g/day and is the result of the weekly weight gain average measured during routine neonate screenings.

The Apgar Scale was described by Virginia Apgar to assess the newborn's overall condition based on heart rate, breathing effort, response to stimuli, muscles tone, and skin color of the newborn. Apgar Scale at minute 1 of birth reflects the newborn's tolerance to birth, and Apgar at minute 5 reflects their adaptability level to the environment (23, 28).

Bronchopulmonary dysplasia was defined as a preterm infant chronical pulmonary disease with limited respiratory function caused by airway immaturity and exposure to high oxygen saturation due to mechanical ventilation (19). Three levels of severity (mild, moderate, and severe) were established based on gestational age (over or under 32 weeks of pregnancy) and ability to breathe room air or require oxygen at a concentration above or below 30% (29).

Presence of neonatal apnea was defined as the absence of respiratory effort for more than 20 s, or for more than 10 s with bradycardia or desaturation in newborns within the first 28 days of life (30).

Anemia and the need for blood transfusions are two conditions that have been related to ROP severity. The literature shows conflicting results. Some studies show that receiving a higher amount of blood supply has no significant impact on ROP severity (31). However, other studies show that receiving a low number of blood transfusions could result in a higher percentage of fetal hemoglobin, instead of adult hemoglobin, which could be a ROP protective factor (32).

Presence or absence of patent ductus arteriosus was confirmed in all of the cases using echocardiography. Rate of spontaneous closure of ductus arteriosus decreases considerably in newborns of <28 weeks and/or <1,000 g at birth, and its presence has been associated with several pathologies, such as ROP (33).

Sepsis was defined as a life-threatening organ dysfunction caused by a dysregulated host response to infection. Organ dysfunction can be identified as an acute change in total SOFA (Sequential Organ Failure Assessment) score ≥2 points consequent to the infection (25).

Necrotizing enterocolitis was defined as an abdominal distention with pneumatosis intestinalis, portal venous gas, or both; or presence of other radiographic signs, such as fixed, dilated intestinal loops and ileus patterns (34). Most published articles show a higher risk of ROP in preterm infants with necrotizing enterocolitis. However, some authors claim that this relation could be caused by the impact of prolonged treatment with oxygen (3).

Presence of cerebral hemorrhage with intraventricular extension diagnosed by a pediatrician or neuropediatrician and confirmed through imaging was included in the study. Severity was classified using the Graeb Score (35). The presence of intraventricular hemorrhage has been associated with severe stages of ROP (36) and as one of the pathologies with higher morbidity and mortality in preterm newborns.

Presence of periventricular leukomalacia was determined using neuroimaging (primarily, ultrasonography, and/or magnetic resonance imaging) (37).

Identified cases included eyes that received treatment for threshold retinopathy of prematurity (ROP) or type 1 prethreshold ROP. Identified controls included other eyes that did not receive treatment for ROP or those presenting progression-free type 2 prethreshold ROP.

### Statistical Analysis

Statistical analysis was performed using the Statistical Package for Social Sciences (SPSS 25.0; IBM Corp. Armonk, NY: IBM Corp). The variables were analyzed descriptively and expressed as means ± standard deviation. Cuantitative variables were compared using Mann-Whitney *U*-test. Qualitative variables were compared using Pearson's χ^2^ test. The predictive risk model was implemented using binary logistic regression between the dependent variable (ROP requiring treatment) and each of the independent variables.

## Results

### Descriptive Analysis

A total of 683 premature newborns were screened, of which 455 were excluded because they did not meet the inclusion criteria. Of the 228 newborns ultimately included in the study, 38 (16.7%) received treatment, while 190 (83.3%) did not.

The mean GA of the study population was 28.83 ± 2.03 weeks; 34.6% of the newborns had a gestational age of 24–27 weeks, 47.4% of 28–30 weeks, and 18% of 31–34 weeks. The lowest GA in the study sample was 24 weeks, while the highest was 34 weeks.

The mean MV time of the entire sample was 9.4 ± 13.64 days; 28.07% of the newborns did not require MV, 42.98% required ≤ 10 days of MV, and 28.95% required > 10 days of MV.

The mean temporal avascular retinal area in the study sample was 3.46 ± 1.63 DD; 40.4% of the newborns had a temporal avascular area of <3 DD, while 37.7% had an avascular area between 3–4 DD and 21.9% had an avascular area of ≥ 5.00 DD (Table 1).

**Table 1:** Descriptive statistics of the study sample, average difference in continuous variables (Mann–Whitney *U*-test) and percentage difference in qualitative variables (Pearson's chi-square test) within treated and untreated ROP groups.

**Variable**	**Total mean ±*SD* or %**	**Untreated**	**Treated**	***p***
Gestational age (weeks)	28.83 ± 2.03	29.10 ± 1.88	27.44 ±1.96	<0.001
	(Min 24.0/Max 34.0)			
Birth weight (g)	1094 ± 268.9	1126.92 ± 257.67	935.16 ± 270.09	<0.001
	(Min 537/Max 1970)			
Duration of mechanical ventilation (days)	9.4 ± 13.64	6.73 ± 8.62	22.74 ± 23.28	<0.001
	(Min 0/Max 100.0)			
Avascular area of the retina (DD)	3.46 ± 1.63	3.13 ± 1.44	5.13 ± 1.53	<0.001
	(Min 1.0/Max 8.0)			
Weight gain in the first 4 weeks (g/day)	12.19 ± 6.03	12.68 ± 6.01	9.70 ± 5.52	0.003
	(Min 0.1/Max 39.7)			
Number of blood transfusions	0.64 ± 0.67	0.58 ± 0.60	0.95 ± 0.89	0.006
	(Min 0/Max 5.0)			
Apgar score (1 min)	-	-	-	0.046
	(Min 0/Max 10)			
Apgar score (5 min)	-	-	-	0.001
	(Min 0/Max 10)			
Presence of bronchopulmonary dysplasia	81.6%	79.5%	92.1%	0.067
Presence of apnoea	14.5%	7.4%	50.0%	<0.001
Presence of persistent arteriovenous ductus	20.6%	15.8%	44.7%	<0.001
Presence of sepsis	40.4%	35.8%	63.2%	0.002
Presence of necrotizing enterocolitis (NEC)	20.6%	18.9%	50.0%	<0.001
Presence of intraventricular hemorrhage	14.0%	10.5%	31.6%	0.001
Presence of periventricular leukomalacia	24.1%	19.5%	47.4%	<0.001

*Min, minimum; Max, maximum*.

### Relationship Between ROP Requiring Treatment and Study Variables

Table 2 shows the results of the binary logistic regression analysis between ROP requiring treatment and the risk factors related to retinal vascular development in ROP identified in the study.

**Table 2:** Analysis of the risk factors for severe ROP requiring treatment (assuming that the other variables remain constant in the sample).

**Variable**	**OR (CI 95%)**	**Nagelkerke's R2**
Avascular temporal retinal diameter (DD)	2.11 (1.64, 2.70)^*^	30.1%^**^
Duration of mechanical ventilation (days)	1.08 (1.05, 1.11)^*^	24.7%^**^
Weight gain in the first 4 weeks (grams/day)	0.90 (0.84, 0.97)^*^	6.5%^**^
Apgar score (1 min)	0.95 (0.83, 1.09)^*^	0.4%^**^
Apgar score (5 min)	0.98 (0.82, 1.17)^*^	0.01%^**^
Gestational age (weeks)	0.63 (0.51, 0.77)^*^	16.1%^**^
Birth weight (g)	0.99 (0.98, 0.99)^*^	13.2%^**^
Degree of bronchopulmonary dysplasia	1.70 (1.21, 2.41)^*^	7.9%^**^
Number of blood transfusions	2.05 (1.22, 3.41)^*^	6.1%^**^
Presence of apnoea	12.57 (5.44, 29.03)^*^	24.5%^**^
Presence of persistent arteriovenous ductus	4.32 (2.04, 9.13)^*^	10%^**^
Presence of sepsis	3.07 (1.49, 6.33)^*^	7%^**^
Presence of necrotizing enterocolitis (NEC)	4.28 (2.06, 8.89)^*^	10.6%^**^
Presence of intraventricular hemorrhage	3.92 (1.72, 8.96)^**^	7.0%^**^
Presence of periventricular leukomalacia	3.72 (1.79, 7.73)^*^	8.6%^**^

* *p < 0.05*,

** *p < 0.001*.

#### Relationship Between ROP Requiring Treatment Retinal Temporal Avascular Area

**Figure 1:**
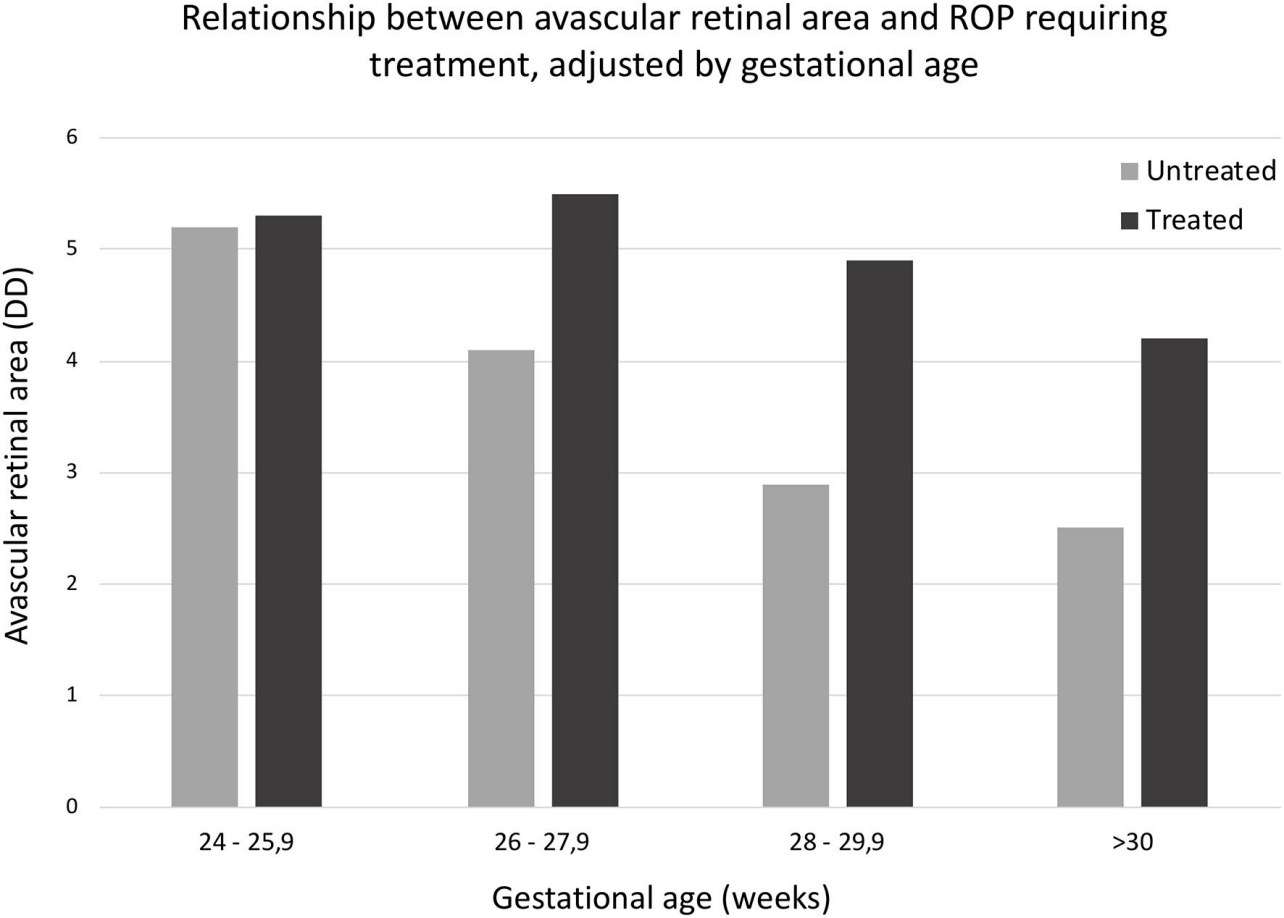
Relationship between retinal avascular area and ROP requiring treatment, adjusted by gestational age. *U* Mann Whitney medium range differences: ROP no requiring treatment, 101.78; ROP requiring treatment, 178.08 (*p* < 0.0001).

Greater retinal avascular area was significantly associated with greater risk of ROP requiring treatment (Nagelkerke's *R*^2^: 30.1%, *p* < 0.0001). The first patients who were treated presented with 2.00 DD without vascularization. Of the patients with ≥ 6 DD of retinal avascular area, 58.83% required treatment (Table 3, Figure 1).

**Table 3:** Relationship between retinal temporal avascular area and ROP requiring treatment in the sample. As avascular area increases, so did the percentage of individuals requiring treatment.

**Retinal avascular area**	**Untreated ROP**	**Treated ROP**	**Total**	**% ROP treated**
1-1.9 DD	20	0	20	0%
2-2.9 DD	69	2	71	2.9%
3-3.9 DD	47	5	52	10.6%
4-4.9 DD	27	8	35	22.8%
5-5.9 DD	13	4	17	23.5%
6-6.9 DD	10	13	23	56.5%
7-7.9 DD	1	4	5	80%
8-8.9 DD	3	2	5	40%

#### Relationship Between ROP Requiring Treatment and Mechanical Ventilation Time

**Figure 2:**
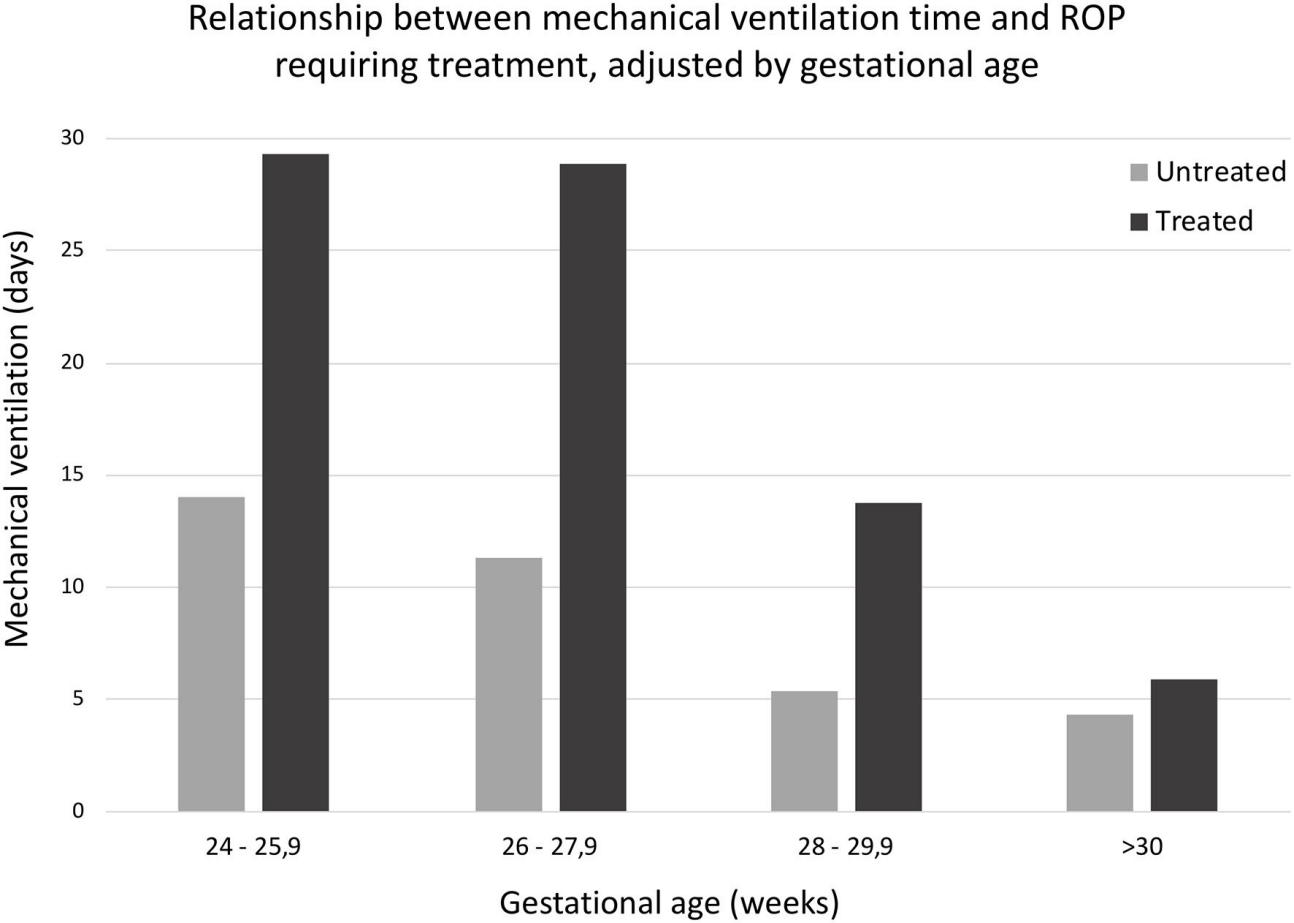
Relationship between mechanical ventilation time and ROP requiring treatment, adjusted by gestational age. *U* Mann Whitney medium range differences: ROP no requiring treatment, 104.98; ROP requiring treatment, 162.09 (*p* < 0.0001).

Longer MV times were significantly associated with greater risk of ROP requiring treatment (Nagelkerke's *R*^2^ = 24.7%, *p* < 0.001), as shown in Table 4. After 31 days of MV, 72.7% of the newborns required treatment (Table 4, Figure 2).

**Table 4:** Relationship between MV time and ROP requiring treatment. As the MV time increases, so does the percentage of individuals treated.

**Mechanical ventilation**	**ROP not treated**	**ROP treated**	**Total**	**% ROP treated**
0 days	59	5	64	7.8%
1–10 days	89	9	98	9.2%
11–20 days	23	9	32	28.1%
21–30 days	16	7	23	30.4%
≥ 31 days	3	8	11	72.7%

### Models for Calculating the Probability of ROP Requiring Treatment

#### Binary Logistic Regression Between ROP Requiring Treatment and Gestational Age at Birth

According to the logistic regression analysis, newborns with lower GA had a greater probability of requiring treatment (Nagelkerke's *R*^2^ = 16.1%, *p* < 0.001; OR: 0.631, 95% confidence interval [CI]: 0.513–0.775, *p* = 0.005]. Applying the logistic regression model between the dependent variable (ROP requiring treatment) and GA in weeks yielded the following:

$$\begin{array}{l} \text{Probability of ROP requiring treatment}\\ \;=1/\left(1+e^{-11.417-0.461\left(\text{weeks of GA}\right)}\right) \end{array}$$

The probability of treatment decreased by 36.9% for each additional week of GA, if the rest of the variables remained constant.

#### Binary Logistic Regression Between ROP Requiring Treatment and Duration of MV

The risk of requiring treatment for ROP is directly proportional to the days of duration of MV (Nagelkerke's *R*^2^: 24.7%, *p* < 0.001) [OR:1.08 (CI: 1.049, 1.115), *p* = 0.005]. As in the previous section, following the logistic regression equations between ROP requiring treatment and the days of duration of MV:

$$\begin{array}{l} \text{Probability of ROP requiring treatment}\\ \;=1/\left(1+e^{-2.571+0.078\left(\text{days of MV}\right)}\right) \end{array}$$

For each additional day of MV, the probability of ROP requiring treatment was increased by 8.1% provided the rest of the variables remained constant.

#### Logistic Regression Between ROP Requiring Treatment and Temporal Avascular Area

A directly proportional relationship was observed between risk of ROP requiring treatment and size of retinal temporal avascular area. That is, larger avascular area was associated with greater risk of requiring treatment (Nagelkerke's *R*^2^ = 30.1%, *p* < 0.001; OR: 2.11, 95% CI: 1.648–2.701, *p* = 0.005). Again, following the logistic regression equations, we concluded that:

$$\begin{array}{l} \text{Probability of ROP requiring treatment}\\ \;=1/\left(1+e^{-4.640+0.746\left(\text{DD of avascular area}\right)}\right) \end{array}$$

For every DD of additional retinal temporal avascular area in the premature newborn, the probability of requiring treatment increased by 111% provided the rest of the variables remained constant.

#### Risk Calculator for ROP Requiring Treatment Based on the Retinal Temporal Avascular Area and MV Time

According to the multiple logistic regression model, longer MV time was significantly associated with greater avascular area and greater likelihood of ROP requiring treatment (Nagelkerke's *R*^2^ = 42.9%, *p* < 0.001; OR for avascular area in DD: 1.986, 95% CI: 1.527–2.582, *p* = 0.005; OR for MV time in days: 1.067, 95% CI: 1.033–1.101, *p* = 0.005).

$$\begin{array}{l} \text{Probability of ROP requiring treatment}\\ =1/\left(1+e^{-5.251+0.686\left(\text{avascularareainDD}\right)+0.064\left(\text{MV time in days}\right)}\right) \end{array}$$

On the basis of the equations of this analysis, we can conclude the results shown in Table 5. An automatic online calculator system for predicting ROP requiring treatment was created (available on: www.roprequiringtreatment.com).

**Table 5:** Table for calculating risk of ROP requiring treatment based on the retinal temporal avascular area in DD and the MV time in days, according to the ET-ROP study (38, 39).

**Mechanical ventilation (days)**	**Avascular area (DD)**										
	**0**	**1**	**2**	**3**	**4**	**5**	**6**	**7**	**8**	**9**	**10**
**0**	0.52%	1.03%	2.03%	3.94%	7.54%	13.93%	24.32%	38.96%	55.90%	71.57%	83.33%
**5**	0.72%	1.41%	2.77%	5.35%	10.09%	18.23%	30.68%	46.78%	63.58%	77.61%	87.31%
**10**	0.98%	1.94%	3.77%	7.22%	13.39%	23.49%	37.87%	54.76%	70.62%	82.68%	90.46%
**15**	1.35%	2.65%	5.12%	9.68%	17.55%	29.71%	45.64%	62.50%	76.80%	86.80%	92.88%
**20**	1.85%	3.61%	6.92%	12.86%	22.67%	36.80%	53.62%	69.66%	82.01%	90.05%	94.73%
**25**	2.53%	4.90%	9.29%	16.90%	28.76%	44.50%	61.42%	75.97%	86.26%	92.57%	96.12%
**30**	3.45%	6.63%	12.36%	21.87%	35.73%	52.47%	68.68%	81.32%	89.63%	94.50%	97.15%

*The percentages in green correspond to those subjects with a <10% risk of requiring treatment, those in yellow correspond to those with a 10–15% risk, and those in red to those with a ≥ 15% risk*.

The risk calculator sensitivity and specificity for predicting severe ROP requiring treatment, based on the levels of probability are shown in Table 6.

**Table 6:** Model sensitivity and specificity for predicting severe ROP requiring treatment, based on the three different levels of probability in Table 5 (low risk—green; moderate risk—yellow; high risk—red).

**Probability**	**Sensitivity**	**Specificity**
<10%	100%	48%
10–14.9%	87%	55%
≥15%	70%	66%

## Discussion

The clinical examinations performed during the present study were carried out by a single pediatric ophthalmologist based on the guidelines of the screening program for ROP in Spain (40). Fundus examination was performed using indirect binocular ophthalmoscopy with scleral indentation, which is currently considered the gold standard technique for complete evaluation of the retina. Unlike other automated techniques such as the use of RetCam®, indirect binocular ophthalmoscopy with scleral indentation enables adequate evaluation of the peripheral retina, which is extremely important in the initial stages of ROP (41–43). However, this method does not allow photographic images to be recorded, as the RetCam® does. This limitation was partially resolved using real time video recordings of the images obtained by the binocular ophthalmoscope.

Examination instruments such as RetCam®, retinoscopy, or wide-field angiography have allowed repeated examination and analysis of retinal images in numerous studies (41–44). However, these techniques are rarely available in hospitals, so the indirect binocular ophthalmoscope continues to be used by numerous specialists. Many previous authors have measured the extent of retinal vascularization in patients with ROP to identify ROP stage and evaluate treatment efficacy (26, 45, 46).

Pediatric ophthalmology experts have a great ability to distinguish vascular dilation, ROP stages in poorly dilated eyes, and color and traction morphology of injuries. Several authors have previously measured the degree of retinal vascularization in patients with ROP to identify the ROP stage and evaluate treatment efficacy (26, 45–47). We believe that, with additional practice, indenting the temporal periphery retina and obtaining the avascular area in the DD observed during each neonatal screening should be easy. This information collected all throughout screening also allowed us to identify another progression marker: vascularization speed. Thus, insufficient vascularization speed of <0.5 DD is an indicator of ROP requiring treatment (48).

In our series, two children treated for ROP (5%) had a birth weight equal or above 1,501 g, or a gestational age of more than 30 weeks, implying that they would not have been detected by any predictive factor model, such as G-ROP (49). Two preterm infants with 2.5 DD of non-vascularized temporal retina were treated for ROP. Retinal vascularization of zone III without prior ROP of zone I or II must be accomplished to be discharged from the screening program (if screener has doubts regarding the zone, confirmatory tests may be justified) (50).

Postnatal weight gain was expressed in g/day and is the result of the weekly weight gain average measured during routine neonate screenings. After a thorough analysis of the literature (7, 10, 51–53), the measurement unit chosen for postnatal weight gain was g/day instead of g/kg/day. Newborns who are underweight at birth have been traditionally associated with a lower weight gain during neonatal period and delays in neurological development. However, a lower birth weight is not a pathogenic factor of retinopathy of prematurity (ROP) in itself, but rather, majority of cases reported in the literature are related to gestational factors (delays in fetal growth from maternal, placental or fetal causes, and prioritization of development of vital organs at the expense of other secondary structures, such as the retina) (54, 55) and newborn comorbidity, which can be the cause of lower birth weight (54, 56, 57).

Risk factors that could be related to or derived from treatment with oxygen (presence/absence of apnea and degree of bronchopulmonary dysplasia, which did not show significant differences between the groups with treated and untreated ROP) were excluded from the predictive model for calculating severe ROP requiring treatment, as these factors could be influenced by duration of mechanical ventilation (19, 29), which is indeed included in the model. It was also decided to exclude neonatal comorbidity (number of transfusions, sepsis, necrotizing enterocolitis, intraventricular hemorrhage, and periventricular leukomalacia) from the model (3, 31, 33, 37). In our study, statistically significant differences were found between the presence of those comorbidity factors and the degree of ROP. However, results described in the literature are heterogeneous, and extrapolating the prediction of this sample to other study populations that showed different results would be difficult. We believe that neonatal comorbidity must be valued individually, and that it is closely related to gestational age and birth weight.

A first model of logistic regression, including avascular area, duration of mechanical ventilation, gestational age and birth weight, was elaborated. Ninety-five percent (95%) confidence intervals deriving from this logistic regression for gestational age and birth weight were 0.74–1.37 and 0.99–1.01, respectively. As confidence intervals include the value 0, these were excluded from the calculator tool due to their low statistical significance value in the predictive model. This lack of significance could be the result of patients already being selected at the beginning of the study based on their gestational age and birth weight, according to the Screening Program for Retinopathy of Prematurity in Spain (40).

In our model, avascular area replaces gestational age given that both variables are strongly correlated, as the avascular area of the first test collects information on the pre and postnatal vascular delay. Thus, the calculator tool was developed based on two variables: avascular area and duration of mechanical ventilation. Gestational age and birth weight are the two most relevant variables considered to select newborns requiring screenings for ROP. Both variables are included in the Screening Program for Retinopathy of Prematurity in Spain (40), and most countries have their own screening criteria (40, 50, 58–60).

However, the calculator tool cannot be used to determine which neonate must be screened, but rather to customize the frequency of ophthalmological checkups. The calculation model's main use is to identify neonates that while participating in the screening program must be subject to more frequent screenings and a more intensified comorbidity control, due to a high risk of severe ROP requiring early treatment; or, neonates that can be subject to less frequent screenings due to low risk of severe ROP.

The simple design of the table based on only two variables allows for a quick and easy observation and interpretation by clinicians during their usual clinical practice, in case of unavailability of electronic device at hand. However, this calculator must be improved in the future as these variables only explain 41% of the outcome and must be validated through studies executed at other centers.

ROP has a hereditary component that ranges between 70 and 73% (61, 62). Furthermore, the vascularization of the choroid, vitreous, and retina occurs alongside the development of nutritional supply to these structures (63). In premature newborns, vascular development of the retina occurs parallel to neurological development (64). Genetics and the environment can interact during pregnancy, birth, or in the postnatal period. However, numerous factors, such as oxygen concentration, can influence vascular delay or inhibition of the retina during phase I of ROP. The resulting retinal ischemia may subsequently contribute to the development of neovessels during phase II of ROP (3).

Michaelson reported that the growth rate of the superficial retinal vessels was 100 μm/day in the human fetus (65). Factors such as low GA and other vascular development inhibitors may lead to a greater avascular area in the temporal retina (5, 48). Premature newborns who present with absence of vascularization in zone I at the first examination have a higher risk of ROP requiring treatment than those who present with absence of vascularization in zone II or III (38).

Various models of ROP screening, such as WINROP (7), CHOP-ROP (66), PINT-ROP (51), NED-ROP (67), and CO-ROP (68), have been proposed to identify newborns at risk of ROP and minimize unnecessary examinations in newborns at low risk. However, ROP cannot always be seen to its fullest extent at the first examination of the newborn (69). Therefore, once newborns are selected to undergo ophthalmological examination, clinicians must take into account the above risk factors, especially the avascular area (in DDs) and the MV time (in days). In so doing, at the first examination they will be able to identify newborns at higher risk of requiring treatment. Therefore, the risk calculation model presented in the present study may be useful for pediatric ophthalmologists during clinical practice.

The objective of this study differs from that of most studies published in the literature in a sense that it is not an ROP or severe ROP screening tool, but rather a tool to be used after the first screening, following the screening criteria of each country. By using the screening criteria of each center or country, newborns that are subjected to serial ophthalmologic screening are identified. In each country or center, screenings are carried out on a weekly or biweekly basis (40, 50), or customized to the severity of each newborn. By applying the model for predicting the risk of severe ROP requiring early treatment on the cohort of neonates, we will be able to adapt the periodicity of checkups.

Each country has its own ROP screening program based on gestational age, birth weight, and neonatal comorbidity, although almost all of them share common characteristics. Gestational age threshold is between week 30 (50, 58) and 32 (40, 59) for most countries, and birth weight is around 1,500 g (40, 50, 58, 59). However, in many developing countries, screening criteria are broader, reaching week 34 of pregnancy and 1,850 g of birth weight (60).

Previous studies carried out at our center on a preliminary patient cohort have shown the usefulness of measuring the vascular/avascular area (5, 48). Thus, vascularization speed from the temporal avascular area to the posterior pole below 0.5 DD/week has been identified as an independent risk factor for severe ROP requiring treatment (5). A study carried out at another center on a different patient cohort analyzed the impact of the risk factors for ROP and the effect of control measures of oxygen saturation levels implemented through the Oxygen With Love (OWL) program. This study revealed that the implementation of the OWL program had a significantly positive impact on the reduction of the number of days of tracheal intubation, and reduction of sepsis rates and extremely low postnatal weight gain (<7 g/day) (6).

Many severe ROP screening or early diagnosis tools have been described in the literature in the past few years. However, most of them are proposed as an initial screening tool to minimize screenings in newborns (7–10, 67, 68). whereas the model described in this study is proposed as a tool to be used on neonates who have already been selected for screenings, following the screening criteria of the country involved–in our case, Spain (40). Neonatal algorithms consider risk factors other than gestational age, postmenstrual age, birth weight, and weight gain. However, when models include ocular fundus data, other risk factors appear, such as pre-plus disease, or prolonged use of mechanical ventilator, which also contribute to a higher risk of guaranteed ROP (70).

The WINROP (Weight, IGF-I, Neonatal, ROP) study was carried out by Hellström et al. (71) to identify preterm newborns with risk of severe ROP early on and avoid unnecessary screenings on other neonates. Initially, this algorithm was based on birth weight, gestational age, and serum insulin-like growth factor-1 levels (IGF1) (7), which were later substituted by weekly weight gain (71, 72). This screening tool, like the calculator proposed by our team, can be checked online.

Another screening model, described with the purpose of identifying newborns that need ophthalmologic screenings due to risk of ROP, is the Children's Hospital of Philadelphia Retinopathy of Prematurity Risk Model. A total of 524 neonates of ≤ 30 weeks of pregnancy (WOP) or <1,501 g of weight were included in this retrospective study to determine, through multivariate logistic regression techniques, the cut-off point of each parameter that allowed an adequate identification of subjects with risk of type 1 or 2 ROP (8, 66).

On the other hand, the ROP Score algorithm, introduced by Eckert, Fortes Filho et al., was based on the aggregate risk of its risk factors. This algorithm was compared with the individual use of birth weight and gestational age in a cohort of 474 neonates of ≤ 1,500 g and/or ≤ 32 WOP. Risk factors included in the algorithm were the following: birth weight, gestational age, postnatal weight gain during the first 6 weeks, treatment with oxygen and need for blood transfusion in the first 6 weeks (9).

The Premature Infants in Need of Transfusion (PINT) study was created to analyze the effect of blood transfusions and level of hemoglobin on the morbidity and mortality of preterm infants (10). However, a model of early diagnosis in newborns with extremely low birth weight was developed from a patient cohort of the PINT study, based on gestational age, birth weight, and daily weight gain (51). The CO-ROP model, developed at the University of Colorado, describes a new ROP screening algorithm based on birth weight, gestational age and postnatal weight gain. The algorithm included newborns of ≤ 30 WOP and ≤ 1,500 g at birth and a net weight gain of ≤ 650 g during the 1st month of postnatal life (68). The Netherlands Retinopathy of Prematurity Study Model (NEDROP) studied several ROP screening strategies and concluded that the strategy that most efficiently diagnosed ROP was the one including newborns of ≤ 30 WOP and ≤ 1,250 g of birth weight and neonates of gestational age between 30–32 WOP and 1,250–1,500 g of birth weight, with at least one risk factor (treatment with oxygen or corticosteroids, sepsis, necrotizing enterocolitis, or heart disease) (73).

Gopal suspects intuitively that the extension of the avascular retina at birth should be a predictive factor for the need for treatment of ROP (74). Meanwhile, Jayadev classifies the temporal avascular area of the retina into three degrees: (a) mild: vessels reach posterior zone III; (b) moderate: vessels enter anterior zone II; (c) severe: vessels in posterior zone I or zone II. Thus, type 1 and type 2 ROP are predicted during the first screening visit (27). Jayadev and we have determined that the avascular area of the retina is the main risk factor for ROP requiring treatment. Jayadev's multivariate analysis also includes gestational age, birth weight and sex, whereas our new model includes duration of mechanical ventilation.

### Limitations of the Study

As previously mentioned, our examination method did not allow quality photographic images to be collected. However, we were able to capture the examination images obtained. The examinations were performed by a single pediatric ophthalmologist during routine clinical practice, and the images or scans were not repeated or analyzed by multiple observers. On the other hand, over the course of the study, from 2009 to 2012, the oxygen therapy protocols underwent a drastic change, because the Oxygen with Love (OWL) program was implemented in the NICU. The application of this program significantly reduced the duration of MV, and therefore the rates of sepsis and ROP (6).

One of the strengths of our study is the wide sampling size, which provides more reliable results. However, one of the limitations is that more studies are needed to confirm the results and validate them in other populations. Additionally, this calculator model does not include every ROP risk factor, for the reasons stated above.

## Conclusion

Premature newborns should be evaluated in a multifactorial manner, taking into account all ROP risk factors, and the neonatal comorbidities such as presence of sepsis, apnea, bronchopulmonary dysplasia, arteriovenous ductus, and blood transfusions. The MV time and the extent of the retinal avascular area in the first examination are crucial for the development and evolution of ROP, so both should be taken into account when predicting ROP severity and the need for treatment. The risk calculator can identify the premature newborns with risk of ROP that requires treatment from their first examination in order to adapt the frequency of examinations according to the level of risk.

The calculator is not a substitute for ROP screening programs or gestational age and birth weight thresholds in each country. It is rather an additional tool to be used for neonates who are already included in the screening to identify those needing closer follow-up or special care. More studies involving a larger number of patients, different centers and a specific cohort of individuals with extremely-low birth weight are needed to confirm and validate these results.

## Data Availability Statement

The datasets generated for this study are available on request to the corresponding author.

## Ethics Statement

The studies involving human participants were reviewed and approved by Biomedical Research Ethics Committee of Andalusia. Written informed consent to participate in this study was provided by the participants' legal guardian/next of kin.

## Author Contributions

All authors have contributed significantly to the work, have read the manuscript, attest to the validity, legitimacy of the data, its interpretation, and agree to its submission to this journal.

## Conflict of Interest

The authors declare that the research was conducted in the absence of any commercial or financial relationships that could be construed as a potential conflict of interest.

## Abbreviations

ROP: retinopathy of prematurity
MV: mechanical ventilation
DD: disc diameters
GA: gestational age
NICU: neonatal intensive care unit.
